# Serum minerals (Ca, P, Co, Mn, Ni, Cd) and growth hormone (IGF-1 and IGF-2) levels in postmenopausal Saudi women with osteoporosis

**DOI:** 10.1097/MD.0000000000020840

**Published:** 2020-07-02

**Authors:** Sobhy M. Yakout, Fatimah Alharbi, Saba Abdi, Nasser M. Al-Daghri, Abir Al-Amro, Malak Nawaz Khan Khattak

**Affiliations:** a Biochemistry Department, College of Science; b Department of Biochemistry, Chair for Biomarkers of Chronic Diseases, College of Science, King Saud University, Riyadh, Saudi Arabia.

**Keywords:** growth hormone, minerals, osteoporosis, postmenopausal, women

## Abstract

Osteoporosis is reported to be common among Saudi women. Several minerals appear to be important determinants of insulin-like growth factor (IGF), the bioactivity of which regulates bone and mineral metabolism. Here we proposed that mineral status may alter the IGF system among individuals with osteoporosis. This study aims to evaluate the relationships between essential elements and IGF levels among postmenopausal Saudi women with osteoporosis. A total of 128 postmenopausal Saudi women aged ≥50 years old were recruited in this study. Diagnosis of osteoporosis was done by using dual-energy x-ray absorptiometry to determine the bone minerals density (BMD). Serum calcium and phosphate were determined using routine chemical analyzer. Serum Co, Mn, Ni, Cd were measured using inductively coupled plasma mass spectrometry. Serum IGF-1 and IGF-2 were determined using Luminex xMAP. Using stepwise linear regression analysis, only Cd was identified to be significantly associated with IGF1 in osteoporosis, explaining 3% (confidence interval 0.01–0.05; *P* = 0001) of the variance perceived. Our results suggest that Cd exposure indirectly affects BMD which may increase the risk of osteoporosis in postmenopausal women. Further longitudinal study using a larger sample size is recommended to determine causality of Cd levels and IGF-1.

## 1 Introduction

Growth hormone (GH) is the most abundant hormone secreted by the hypophysis. Containing a 191-amino-acid polypeptide, GH is important not only in growth action but also in carbohydrate and lipid metabolism. GH has a several effects on different target tissues and cells, which include bone, cartilage, adipose tissue, muscle, heart, and the immune system cells.^[1]^ Insulin-like growth factor (IGF) contains IGF-1 and IGF-2, polypeptide hormones which are similar to insulin.^[2]^ IGFs-1 and 2 play an important role in growth, cellular metabolism and differentiation.^[3]^

There are several hormones and growth factors that regulate mineral and bone metabolism. GH stimulates bone turnover as it upregulates osteoblast production through IGF-1 and IGF-2 synthesis.^[4]^ Also, it stimulates bone resorption through still unknown mechanisms.^[5]^ IGF-1 increase the levels of renal 25-hydroxyvitamin D and consequently improves intestinal phosphate and calcium.^[6]^ Moreover, IGF-1 increases phosphate reabsorption at the renal tubules.^[6]^ The influence of GH on bone looks useful as bone mass is increased together with the lean body mass.^[5]^ Excess GH also increases risk of osteoarthritis and other serious metabolic side effects, therefore its role in the treatment and the prevention of osteoporosis needs to be defined.^[4]^

Essential elements play an important role in several processes necessary for life by mediating vital biochemical reactions as cofactors for many enzymes and proteins structures.^[7]^ At the cellular level, essential elements are important for the stabilization of the cellular structures, but in deficient states they could stimulate alternate pathways and cause diseases.^[7]^ Excessive levels of these elements can be toxic for the human body. Recently, efforts have been made to focus on understanding the relationship between essential elements and many diseases.^[7]^

Our knowledge of the abundance of trace elements in human tissues and fluids has increased significantly due to improvements in the analytical methods, which have become a useful approach in exploring the relationship between basic composition of body fluids and tissues, pathological conditions, and general nutritional status.^[8–10]^ However, evidence on essential element status and its relation to IGF-1 and IGF-2 among individuals at risk for osteoporosis, such as postmenopausal women, is scarce. This study was designed to evaluate the relationships between essential elements and IGF levels among postmenopausal Saudi women with osteoporosis, considered first in the Middle East region.

## 2 Methodology

### 2.1 Subjects

In this cross-sectional study, the samples used were collected from the master database of the Chair for Biomarkers of Chronic Diseases (CBCD), College of Science, King Saud University (KSU) in Riyadh, Saudi Arabia. Samples were previously collected by trained researchers in several hospitals around Riyadh as part of the osteoporosis registry. These hospitals include, but not limited to, King Fahad Medical City (KFMC), King Khalid University Hospital (KKUH) and King Salman Hospital (KSH), where the bulk of the recruitment was done. The study began in early 2013 and the recruitment ended in August 2016. Out of the 5000 plus cases gathered, a total of 128 samples from Saudi postmenopausal women aged ≥50 years old (N = 68 with osteoporosis and N = 50 without osteoporosis) were randomly selected. All measurements were performed systematically between August 2013 and September 2014. The subjects’ information was taken from a generalized questionnaire including age, the age of menarche, the age of menopause, family history for osteoporosis, medical history, disease status, etc. Participants with acute medical conditions that require immediate medical attention and with other associated diseases and inflammatory condition were excluded. The study was approved with number 8\25\454266 by the Ethics Committee of the College of Science, King Saud University, Riyadh, Kingdom of Saudi Arabia.

### 2.2 Anthropometry and blood collection

All anthropometric measurements were made according to the WHO recommendations as previously conducted.^[11,12]^ Anthropometry included height (cm) and weight (kg) which were measured using standardized methods, with the participant wearing light clothes without shoes. Waist (cm) and hip circumferences (cm) were measured using a standardized non-stretchable fiber measuring tape, Waist-hip ratio (WHR) was calculated as the ratio of waist and hip circumferences. Systolic and diastolic blood pressure (mmHg) was measured using mercurial sphygmomanometer. Body mass index (BMI) was calculated as weight (kg) divided by height in squared meters (kg/m^2^).^[13]^ Overnight fasting blood samples (5 ml) were collected from each individual and processed for separation of serum samples. The remaining blood and serum samples were transported to CBCD, KSU, Riyadh, KSA in specialized containers for biochemical analyses and storage at −80°C.

### 2.3 Determination of bone mineral density (BMD)

BMD (g/cm^2^) was measured for femoral neck by dual-energy X-ray absorptiometry DXA (Hologic QDR 2000 Inc., Waltham, MA). The diagnostic criteria of osteoporosis based on the T-score for BMD established according to The World Health Organization definitions that use T-score assessment, T-score value above − 1indicate Normal, T-score between −1 and −2.5 indicate Osteopenia, T-score below −2.5 indicate osteoporosis.

### 2.4 Biochemical analyses

All biochemical assays were done in CBCD, KSU, and KSA. Fasting serum samples were analyzed for blood lipid profile, glucose, calcium, and phosphorous in all participants using routine chemical analyzer (Konelab20XTi, Thermo Electron Corporation, Vantaa, Finland). Serum calcium and Pi were assessed using a chemical analyzer (Konelab, Espoo, Finland) kits purchased from Thermo Fisher Scientific Oy, ref 981367. Calcium, intra- and inter-assay CV were 0.2% and 0.1%, respectively. Pi, intra- and inter-assay CV was 1.9% and 4.7%, respectively.

### 2.5 Luminex assays

The Luminex kits were obtained from Millipore (Billerica, MA) and assays were conducted as done previously^[14]^ to determine serum levels of IGF-1, IGF-2, IL-6, and TNF-α proteins. Properly diluted serum samples were incubated with the antibody-coupled microspheres and then with biotinylated detection antibody before the addition of streptavidin-phycoerythrin. The captured bead complexes were measured with FLEXMAP 3D system (Luminex Corporation, Austin, TX) using the following instrument settings (events/bead, 35; sample size, 50 mL; discriminator gate, 8000–15,000). The raw data (mean fluorescence intensity) were collected and further processed for calculating protein concentration.

### 2.6 Serum Co, Mn, Ni, Cd levels determination.

Serum Co, Mn, Ni, Cd levels were measured using Inductively Coupled Plasma-Mass Spectrometry (ICP-MS). Samples were prepared for ICP-MS analysis by adding 150 μl of each serum sample to a tube, then adding 150 μl of nitric acid and 100 μl of hydrogen peroxide, centrifuged for 10 minutes at speed of relative centrifugal force (rcf) = 2125 × g (Sigma Laborzentrifugen GmbH, Germany). The tubes were then placed in block digester (Thermoblock, Biometra, Analytik Jena, Germany) and digested at 95°C for 90 minutes. The volume was completed with 1 ml with distilled water and stored at 4°C. The analyte concentration of each sample was calculated automatically using calibration curve for each element in ICP-MS.

### 2.7 Statistical analysis

Data were analyzed using statistical package software SPSS (version 21, IBM). Continuous data were presented as mean ± standard deviation (SD) for variables following Gaussian variables and Non-Gaussian variables were presented in median (1st and 3rd) percentiles. All categorical variables were presented in percentages (%) and checked association using the chi-square test. All continuous variables were checked for normality using Kolmogorov-Smirnov test if not normal then Non-Gaussian variable transform to log transform. Independent t-test in and Mann Whitney U were used to check mean and median difference for Gaussian and non-Gaussian variables even it transforms but again it is not normal and adjusted for age and BMI. Correlations between variables were done using Pearson's and Spearman correlation analysis. All figures were plotted in MS Excel. *P* value < .05 was considered statistically significant.

## 3 Results

The general characteristics of 128 postmenopausal Saudi women with and without osteoporosis as shown in Table 1. The osteoporosis group (58.6 ± 6.9 years) was significantly older than those without osteoporosis (50.4 ± 6.5 years). Results were therefore adjusted for age, and the p-value was calculated before and after this adjustment. The mean BMI was not significantly different in both groups. After adjusting for age, significant differences were seen between groups with respect to levels of Ca, Mn, Cd and IGF-2 (*P* values = .04, .007, .005, and .001, respectively), all of which were higher in the osteoporosis group. All the other measured parameters showed no statistically significant difference between groups.

**Table 1:**
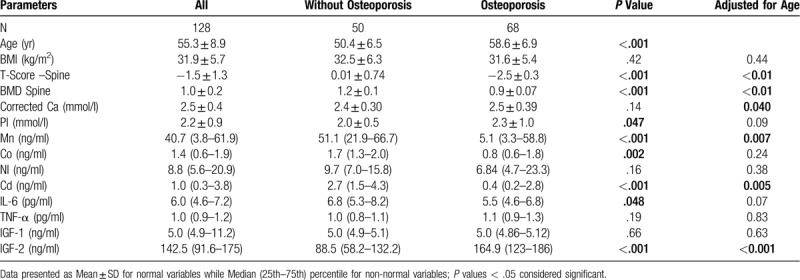
Clinical characteristics of the subjects.

The correlations of IGF-1 and IGF-2 with other parameters are shown in Table 2. In all subjects, IGF-2 was associated with age and was inversely associated with T-Score and BMD as well as Mn and Cd. However, no associations were observed with IGF1. In the osteoporosis group, IGF-1 was significantly associated with Mn, Ni, and Cd and was inversely associated with T-Score. IGF-2 was significantly associated with T-Score. No associations were observed among those without osteoporosis with respect to IGF-1 and IGF-2.

**Table 2:**
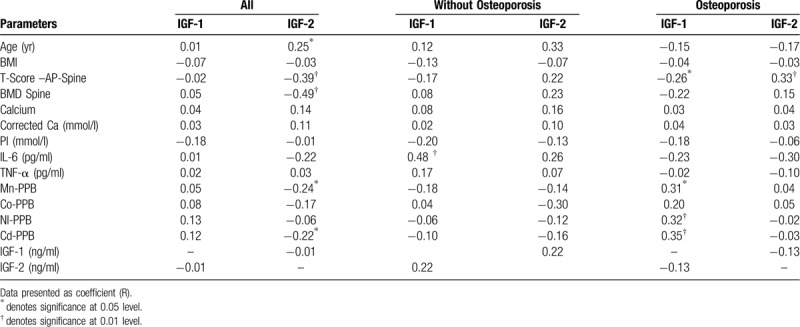
Correlation of IGF-1 and IGF-2 with other parameters.

Table 3 shows the linear regression analysis for IGF-1 and IGF-2 with minerals. Using regression analysis, PI and corrected calcium were observed to be significant predictors for IGF-1. Stratified according to the presence of osteoporosis, almost all minerals were associated with IGF1 with the exception of Ni. No significant predictors were produced in IGF2- in all subjects and after stratification.

**Table 3:**
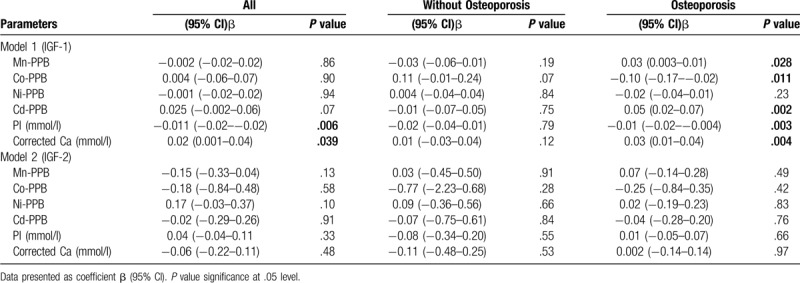
Linear regression analysis (enter) for IGF-1 and IGF-2 (dependent variable with minerals).

Table 4 shows the stepwise linear regression analysis with minerals as independent variables. Cd, PI and corrected Ca were observed to be significant predictors for IGF-1 in all subjects. On the other hand, only Mn was observed to be significant predictors for IGF-2 in all subjects. Stratified according to the presence of osteoporosis, only Cd was associated with IGF-1 in the osteoporosis group. No significant predictors were produced in IGF-2.

**Table 4:**
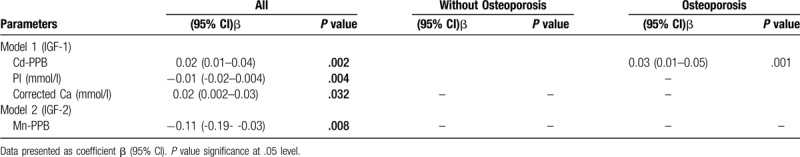
Stepwise linear regression analysis for IGF-1 and IGF-2 (dependent variable with trace minerals).

**Figure 1:**
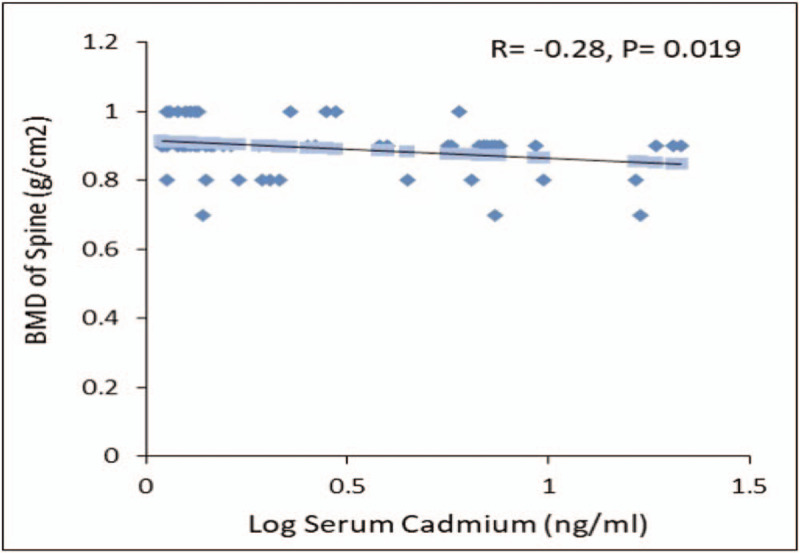
Inverse association between serum cadmium and BMD in osteoporosis group.

In the osteoporosis group, Cd showed a significant inverse correlation with BMD of the spine (R = −0.28, *P* = .019) as seen in Figure 1. Furthermore, area under the curve using Cd concentration to detect osteoporosis was 0.74 (0.65–0.84) (*P* < .001). The sensitivity, specificity, negative predictive, and positive predictive values were 71.4%, 77.3%, 65.4, and 81.8 respectively when cadmium level ≤1.385 was used to diagnose osteoporosis against the gold standard of BMD classification.

## 4 Discussion

Our findings showed a strong significant association between Cd levels and IGF-1 in osteoporosis subjects. Our results support the suggestion that Cd exposure affects BMD which may increase the risk for osteoporosis in postmenopausal women. These result is supported by several studies in Belgium,^[15]^ Sweden,^[16]^ Japan,^[17]^ and China.^[18]^ A study on Saudi women found that women with blood Cd ≥ 0.627 μg/L were 4 times more likely to be hypertensive than those with blood Cd levels < 0.627 μg/L.^[17]^ In 2003, the same authors reported the presence of Cd in breast milk.^[18]^ Widespread environmental pollutants and food is the largest source of Cd exposure in the general population.^[19,20]^

There are limited studies that investigated the relation between Cd and IGF-1. Although some studies have shown a positive relationship between them, others have indicated that the relationship is not significant.^[21]^ A similar study found a proportional relationship between Cd and IGF-1 levels, with higher Cd levels at high IGF-1 levels.^[22]^ The relationship of blood Cd with IGF-1 levels was also observed among Cd-exposed workers.^[23]^

The effects of Cd on bone can be explained by both direct and indirect mechanisms. The direct mechanism is by acting on osteoblasts or osteoclasts, and thus inhibiting bone formation and stimulating bone resorption.^[25]^ Cd-induced increase in the activity of caspase-3, a key protease in apoptosis may cause osteoblasts death.^[24]^ and the indirect mechanism is by interfering with calcium, vitamin D, and renal dysfunction.^[25]^ In the present study, we found that Cd was interfering with calcium metabolism and thus may be indirectly enhancing bone mineral loss.

It has been shown in several experimental studies that IGF-1 increases the activity of 1 α hydroxylase which transforms the inert 25-hydroxyvitamin D into its active form 1,25-dyhydroxyvitamin D.^[26–30]^ Active vitamin D is a well-known regulator of serum calcium. IGF-1 effects on bone resorption and bone formation.^[5]^ This also suggests a potential interaction between calcium and IGF-1 in terms of bone metabolism. Cd is one of the heavy metals physiochemical properties very similar to that of calcium (Choong et al., 2014).^[31]^ Cd exists as the Cd^2+^ ion in biological systems, and in this state structurally resembles Ca^2+^. It is now well appreciated that Cd^2+^ participates in a number of Ca^2+^-depenendent pathways, attributable to its actions as a Ca^2+^ mimetic.^[31]^ This naturally results in an exchangeability of the 2 ions in Ca^2+^ binding protein like calmodulin, sarcolemma and troponin C.^[32]^ IGF-1 induces a rapid increase in calcium currents and spontaneous membrane activity in clonal pituitary cells.^[6]^ Cd interacts with receptors and ion channels on the cell surface, and with the intracellular estrogen receptor where it binds competitively to residues shared by Ca^2+^.^[29]^

The authors acknowledge several limitations. The cross-sectional design limits the findings to at least suggestive. The sample size was small and as such some associations maybe subject to type 2 error. The study nevertheless is the first of its kind in the Middle East to link levels of Cd to osteoporosis risk among postmentopausal women.

## 5 Conclusion

Cd exposure may affect BMD and as such may increase the risk to osteoporosis in postmenopausal women in Saudi Arabia. Osteoporosis health programs should focus on increasing public awareness to prevent unnecessary Cd exposure. There is an association between Cd status and IGF-1 status, although the nature of this relationship and the underlying mechanism remains unclear. IGF-1 may be used as biomarker for the early detection of osteoporosis caused by Cd exposure and should be taken into consideration in investigations of such population. Further, longitudinal study on larger number of subjects is needed to determine the causality of Cd status and IGF-1 relationships and before we could substantiate our findings to the whole population.

## Acknowledgments

The authors highly acknowledge the participation of the individuals in their study and they are also highly grateful to the Chair for Biomarkers of Chronic Diseases, Biochemistry Department, College of Science, King Saud University, and Deanship of Scientific Research for the technical support.

## Author contributions

**Conceptualization:** Fatimah Alharbi.

**Formal analysis:** Malak Nawaz Khan Khattak.

**Investigation:** Sobhy Yakout, Saba Abdi.

**Methodology:** Fatimah Alharbi, Abir Al-Amro, Malak Nawaz Khan Khattak.

**Supervision:** Saba Abdi, Abir Al-Amro.

**Writing – original draft:** Sobhy Yakout.

**Writing – review & editing:** Fatimah Alharbi, Saba Abdi, Abir Al-Amro, Malak Nawaz Khan Khattak.
